# Narrative Imagination: Dismantling Old Age Through The Literary Foodscape In Lore Segal's "The Arbus Factor"

**DOI:** 10.1093/geroni/igab046.2295

**Published:** 2021-12-17

**Authors:** Eva-Maria Trinkaus

**Affiliations:** University of Graz, Graz, Steiermark, Austria

## Abstract

Being able to take another person's perspective and understanding the Other is a crucial element of reading, understanding, and processing literature. Especially in the context of old age, many literary texts play into the culturally constructed (cf. Gullette 2004) and biased understanding of old age as decline narrative, rather than reading an old person's story as a narrative of possibility. In her short story "The Arbus Factor" which was first published in The New Yorker in 2007, Lore Segal offers a different perspective on aging. Through creating a space, coming into existence through foodways and food practices, which in my dissertation I will refer to as 'literary foodscape,' she offers a setting and backdrop for the characters to construct a discourse of possibility, creation, and new opportunities at a later stage in life. Segal wittily dismantles age-related stereotypes and opens up a discourse that goes beyond an easy categorization. This paper is going to analyze the ways in which a literary text, through the 'literary foodscape' is able to rewrite a culturally engrained perspective, and offers a different and more accurate understanding of what it means to be old. Gullette, Margaret Morganroth. Aged By Culture. The University of Chicago Press. 2004.

